# Sexual Coercion by Women: The Influence of Pornography and Narcissistic and Histrionic Personality Disorder Traits

**DOI:** 10.1007/s10508-019-01538-4

**Published:** 2019-10-07

**Authors:** Abigail Hughes, Gayle Brewer, Roxanne Khan

**Affiliations:** 1 School of Psychology, University of Central Lancashire, Preston, Lancashire PR1 2HE UK; 2 School of Psychology, University of Liverpool, Liverpool, UK

**Keywords:** Female perpetration, Histrionic personality traits, Narcissistic personality traits, Sexually explicit material

## Abstract

Largely overlooked in the literature, this study investigated factors influencing women’s use of sexual coercion. Specifically, pornography use and personality disorder traits linked with poor impulse control, emotional regulation, and superior sense of sexual desirability were considered. Women (*N* = 142) aged 16–53 years (*M* = 24.23, *SD* = 7.06) were recruited from community and student populations. Participants completed the Narcissistic and Histrionic subscales of the Personality Diagnostic Questionnaire-4, in addition to the Cyber-Pornography Use Inventory to explore the influence of their pornography use (interest, efforts to engage with pornography, and compulsivity) on their use of sexual coercion. This was measured using four subscales of the Postrefusal Sexual Persistence Scale: nonverbal sexual arousal, emotional manipulation and deception, exploitation of the intoxicated, and use of physical force or threats. Multiple regression analyses revealed that pornography use, narcissistic traits, and histrionic traits significantly predicted the use of nonverbal sexual arousal, emotional manipulation and deception, and exploitation of the intoxicated. Effort to engage with pornography was a significant individual predictor of nonverbal sexual arousal and emotional manipulation and deception, while histrionic traits were a significant individual predictor of exploitation of the intoxicated. Findings were discussed in relation to existing sexual coercion literature and potential future research.

## Introduction

Sexual aggression research has historically focused on male perpetration and female victimization. This approach most likely reflects the global pervasiveness of men’s sexual violence and perceptions of women as sexually passive (Denov, 2017; Krahé & Berger, 2013). However, females also sexually aggress against unwilling partners (Erulkar, 2004; Hines, 2007) and researchers have increasingly acknowledged nuances in how this might be expressed (e.g., by harassment, abuse, and coercion) (Grayston & De Luca, 1999; Ménard, Hall, Phung, Ghebrial, & Martin, 2003). Despite this, and the negative physical and psychological consequences experienced by male victims (Visser, Smith, Rissel, Richters, & Grulich, 2003), a dominant gendered perspective has resulted in a relative paucity of information on factors that may explain female sexual aggression (Campbell & Kohut, 2017; Denov, 2017). This area is worthy of investigation as pathways to sexual aggression differ for men and women (Krahé & Berger, 2017), and factors associated with sexual coercion by men may not be generalizable to female perpetrators. Indeed, Schatzel-Murphy, Harris, Knight, and Milburn (2009) found that while men and women’s sexually coercive behavior may be similar, factors symptomatic of its use might be different, with sexual compulsivity (i.e., difficulty controlling sexual urges) shown to be a dynamic influence for females. Our study, therefore, aimed to investigate factors associated with sexual compulsivity in women that might explain their use of sexually coercive behavior. Specifically, the influence of three elements of pornography use (interest, efforts to engage with pornography, and compulsivity) and narcissistic and histrionic personality traits was explored due to associations in the literature with coercive sexual tactics to obtain intimate relations.

Sexual coercion lies on the sexual aggression continuum and is defined as “the act of using pressure, alcohol or drugs, or force to have sexual contact with someone against his or her will” (Struckman-Johnson, Struckman-Johnson, & Anderson, 2003, p.76). Sexual coercion may include a range of behaviors that can be separated into four categories of increasing exploitation: (1) sexual arousal (e.g., persistent kissing and touching), (2) emotional manipulation (e.g., blackmail, questioning, or using authority), (3) alcohol and drug intoxication (e.g., purposefully getting a person drunk or taking advantage while intoxicated), and (4) physical force or threats (e.g., using physical harm). As a large body of research has established that men are more likely than women to perpetrate sexual coercion (see Krahé et al., 2015), this has overshadowed evidence that a proportion of women also report using a range of sexually coercive behavior (e.g., Hoffmann & Verona, 2018; Krahé, Waizenhöfer, & Möller, 2003; Ménard et al., 2003; Muñoz, Khan, & Cordwell, 2011; Russell & Oswald, 2001, 2002; Struckman-Johnson et al., 2003). While single studies have found female perpetration rates as high as 26% (compared to 43% for males) (see Struckman-Johnson et al., 2003), in an overview of the literature, Hines (2007) estimated rates between 10 and 20% for verbal sexual coercion, and 1 and 3% for physically forced sexual intercourse.

Due to higher rates of male perpetration, it is perhaps not surprising that fewer studies have focused on correlates of women’s sexually coercive behavior. Studies have reported that influential factors for women include peer pressure to have sex (e.g., Krahé et al., 2003), sexual compulsivity (Schatzel-Murphy et al., 2009), antagonistic attitudes toward sexual relationships (e.g., Anderson, 1996; Christopher, Madura, & Weaver, 1998; Yost & Zurbriggen, 2006), and sexual victimization experiences (e.g., Anderson, 1996; Krahé et al., 2003; Russell & Oswald, 2001). Further studies have documented the influence of a hostile personality with a dominant interpersonal style (Ménard et al., 2003) a manipulative, game-playing approach to forming intimate relations (Russell & Oswald, 2001, 2002), and pornography use (e.g., Kernsmith & Kernsmith, 2009a) thereby providing the rationale for this study.

### Women’s Use of Pornography

Pornography refers to sexually explicit material developed and consumed to stimulate sexual arousal, available in versatile forms (e.g., photographs and videos) and often accessed online (Campbell & Kohut, 2017). Research has historically focused on the manner in which exposure to pornographic material influences men’s sexual attitudes and conduct. For example, it is argued that men’s use of pornography is related to sexual objectification of partners (Tylka, & Kroon Van Diest, 2015) and sexually coercive behavior (Stanley et al., 2018). Compulsive consumption of pornographic material, in particular, may be closely related to men’s sexually aggressive behavior (Gonsalves, Hodges, & Scalora, 2015). Research indicates that women also engage with pornography, although to a lesser extent than men (Ashton, McDonald, & Kirkman, 2018; Rissel, Richters, de Visser, McKee, Yeung, & Caruana, 2017). Due to disparities in methodology, estimates of women’s pornography use vary significantly across studies, ranging from 1 to 88% depending on the sample and operational definition of pornography (Campbell & Kohut, 2017). In a review of their annual statistics, Pornhub, a large Internet pornography website, reported that just over a quarter of their visitors were women and that their top trending^1^ search throughout 2017 was “porn for women,” representing a 1400% increase (Pornhub Insights, 2018). While some studies report that females were more likely to use pornography with a partner (e.g., Ševčíková & Daneback, 2014), other studies have found that their pornography use was more likely and more frequent when alone than with a partner (Fisher, Kohut, & Campbell, 2017).

Consistent with studies of men’s pornography consumption, research has found women’s use of pornography to be associated with attitudes toward sex, sexual conduct, and sexual activities (e.g., number of sexual partners) (Wright, Bae, & Funk, 2013). This is supported further by a recent meta-analysis that found, similar to men, women’s pornography use was associated with sexual aggression, both verbally (i.e., “verbally coercive but not physically threatening communication to obtain sex, and sexual harassment”) and physically (i.e., “use or threat of physical force to obtain sex”) (Wright, Tokunaga, & Kraus, 2016, p.191). The small number of studies in this area has meant the extent to which women’s use of pornography influences their sexually aggressive behavior remains unclear. In one such study, it was found that pornography use predicted all forms of sexual aggression in women (i.e., extortion, deceit, obligation, and emotional manipulation) except for physical violence and intimidation (Kernsmith & Kernsmith, 2009a). The dearth of literature available indicates there is scope to investigate this further, thus we consider three elements of women’s pornography use, that is (1) interest in pornography, (2) efforts to engage with pornography, in additional to (3) pornography compulsivity, which is largely overlooked despite its association with men’s sexual aggression (e.g., Gonsalves et al., 2015).

### Narcissistic and Histrionic Personality Disorder Traits

Personality traits may also influence the likelihood of sexually aggressive behavior in women (Krahé et al., 2003; Russell, Doan, & King, 2017). Characteristics of the dramatic, emotional, and erratic Cluster B personality disorders (associated with poor impulse control, emotional regulation, and anger) may be particularly influential on sexual aggression (Mouilso & Calhoun, 2016). For example, narcissistic personality disorder (NPD), found in both men (7.7%) and women (4.8%) and overall in 6.2% of the general population (Stinson et al., 2008), is characterized by a grandiose sense of the self, entitlement, and low empathy for others (Emmons, 1984). In men, narcissistic personality traits are positively associated with rape supportive beliefs and negatively associated with empathy for rape victims (Bushman, Bonacci, van Dijk, & Baumeister, 2003), while NPD is related to perpetration of sexual aggression (Mouilso & Calhoun, 2016). Women with higher levels of narcissism display more negative relationship communication (Lamkin, Lavner, & Shaffer, 2017) and are more likely to engage in sexual harassment (Zeigler-Hill, Besser, Morag, & Campbell, 2016). Pertinently, narcissism is associated with women’s perpetration of sexual coercion (Kjellgren, Priebe, Svedin, Mossige, & Långström, 2011; Logan, 2008), with the entitlement/exploitativeness dimension found to be most influential (Blinkhorn, Lyons, & Almond, 2015; Ryan, Weikel, & Sprechini, 2008). Additionally, females high in narcissism were found to be just as likely as their male counterparts to react with persistence and sexually coercive tactics after being denied during a sexual advance (Blinkhorn et al., 2015). In part, this behavior may reflect the tendency for narcissistic individuals to engage in sex in order to fulfill their need for self-affirmation (Gewirtz-Meydan, 2017).

Found in 1–3% of general population (Torgersen et al., 2000) and reported twice more in women than in men (Torgersen, Kringlen, & Cramer, 2001), traits associated with histrionic personality disorder (HPD) are far less explored than NPD in relation to sexual coercion. This is somewhat surprising as defining characteristics of HPD include excessively emotional, impulsive, attention seeking behavior, and inappropriate or competitive sexual conduct (APA, 2013; Dorfman, 2010; Stone, 2005). Emotionally manipulative and intolerant of delayed gratification (Bornstein & Malka, 2009; Stone, 2005), women with HPD demand confirmation and attention from intimate partners (AlaviHejazi, Fatehizade, Bahrami, & Etemadi, 2016). A study that compared women with HPD to a matched control group without personality disorders found they were more likely to have been sexually unfaithful and report greater sexual preoccupation and sexual boredom with lower levels of sexual assertiveness and relationship satisfaction (Apt & Hurlbert, 1994). Furthermore, Apt and Hurlbert considered that HPD behavioral traits were indicative of sexual narcissism, while Widiger and Trull (2007) noted that HPD and NPD traits were likely to co-occur. The dominant, manipulative, and sexually compulsive behavioral traits found in these studies of women with NPD and HPD are pertinent as they align with extant studies reporting factors underpinning women’s perpetration of sexual coercion (e.g., Russell & Oswald, 2001, 2002; Schatzel-Murphy et al., 2009) and pornography use (e.g., Wright et al., 2013, 2016). Hence, additional research is necessary to examine the influence of both HPD and NPD traits and pornography use on women’s use of sexual aggression.

### Research Aims

This study investigated the influence of pornography use and narcissistic and histrionic personality traits on four types of sexual coercion. In line with past research, we predicted that pornography use (e.g., Kernsmith & Kernsmith, 2009a; Wright et al., 2016) and narcissistic and histrionic personality traits (e.g., Apt & Hurlbert, 1994; Blinkhorn et al., 2015; Kjellgren et al., 2011; Logan, 2008; Ryan et al., 2008) would be significantly associated with greater incidence of three types of sexual coercion (i.e., nonverbal sexual arousal, emotional manipulation and deception, and exploitation of the intoxicated). We also predicted that pornography use and personality traits would not be associated with the use of a fourth type of sexual coercion (i.e., physical force or threats) as this has not been reported in the previous research.

## Method

### Participants and Procedure

A total of 142 women, aged 16–53 years (*M* = 24.23, *SD* = 7.06), participated in this study. Women were typically in a long-term relationship, of at least 6 months duration (*n* = 53.5%). The remaining participants were single or divorced (*n* = 24.7%), in a short-term relationship (*n* = 11.3%), or married (*n* = 10.6%). Most participants were heterosexual (*n* = 85.2%), with a smaller number of bisexual (*n* = 11.3%) and homosexual (*n* = 3.5%) women recruited. Just under a half (*n* = 43%) of these women reported that they currently used pornography. No other demographic data were collected. Two modes of opportunity sampling were used to collect information from a diverse sample of women aged over 16 years, in a student and community population, with no known offending history. Participants volunteered to complete either a paper or online questionnaire, estimated to take 15 min. Remuneration was not offered for taking part in this study.

Participants were recruited via undergraduate and postgraduate classes plus recreational spaces within a large university in England, as well as in the local community, inside shopping centers (*n* = 37). The first author distributed questionnaire booklets to potential participants placed inside a self-addressed envelope, to ensure confidential and anonymous return. To obtain informed consent, potential participants were verbally informed of the anonymous and voluntary nature of the questionnaire, which was reiterated on a briefing sheet attached to the questionnaire. This briefing sheet also made clear that questionnaires should be completed alone and that return of questionnaires indicated consent for information to be used. On campus, participants were told they could place completed questionnaires in envelopes to return either to the researcher by hand or to a secure drop-in box in a student resource room. Participants were also recruited via snowballing methods using social media postings on Facebook and Twitter (*n* = 108). These posts detailed the study’s aims and invited females to participate by clicking on a hyperlink that redirected them to view the questionnaire online, so it could be completed securely and remotely.

### Measures

#### Sexual Coercion: Postrefusal Sexual Persistence Scale (PSP Scale, Struckman-Johnson et al., 2003)

The PSP Scale is a 19-item measure of postrefusal sexual persistence, defined as pursuing sexual contact with a partner after they initially refused. The scale is separated into four sections reflecting different levels of sexual exploitation: (1) nonverbal sexual arousal tactics (three items, e.g., “Persistent kissing and touching”); (2) emotional manipulation and deception strategies (eight items, e.g., “Threatening to break up”); (3) exploitation of the intoxicated (two items, e.g., “Purposely getting them drunk”), and (4) use of physical force or threats (six items, e.g., “Tying them up”). Items were scored 1 (yes) or 0 (no), with higher scores indicating greater use of sexual coercion. The internal reliability for each subscale has been mixed in previous studies (e.g., Khan, Brewer, Kim, & Centifanti, 2017), which was reflected in this study: nonverbal sexual arousal (*α* = .81); emotional manipulation and deception (*α* = .39); exploitation of the intoxicated (*α* = .38); and the use of physical force or threats (*α* = .00).

#### Pornography Use: Cyber-Pornography Use Inventory (CPUI, Grubbs, Sessoms, Wheeler, & Volk, 2010)

Three CPUI subscales were employed: interest (two items, i.e., “I have some pornographic sites bookmarked” and “I spend more than 5 h per week using pornography”), efforts to engage with pornography (five items, e.g., “I have rearranged my schedule so that I would be able to view pornography online without being disturbed” and “I have refused to go out with friends or attend certain social functions to have the opportunity to view pornography”), and compulsivity (11 items, e.g., “When I am unable to access pornography online, I feel anxious, angry, or disappointed” and “I feel unable to stop my use of pornography”). One final item “I believe I am addicted to Internet pornography” was not included due to the controversial nature of the terms “sexual addiction” and “pornography addiction” (Schneider, 1994). On the interest and effort subscales, participants indicated responses as “true” (scored 2) or “false” (scored 1), while on the compulsivity subscale, responses were recorded on a 7-point scale (1 = strongly disagree to 7 = strongly agree), with higher scores indicating a greater degree of pornography interest, effort, and compulsion. Reliabilities were: interest *α* = .40; effort *α* = .58; and compulsivity *α* = .75.

#### Narcissistic and Histrionic Personality Disorder Traits: Personality Diagnostic Questionnaire, 4th Edition (PDQ-4: Hyler, 1994)

Items in the PDQ-4 Narcissistic and Histrionic subscales are based on the DSM-IV diagnostic criteria for Axis II disorders and have been used in comparable studies to explore personality disorder traits and use of sexual coercion in females (e.g., Khan et al., 2017; Muñoz et al., 2011). Scores on the Narcissistic subscale (nine items, e.g., “Some people think that I take advantage of others”) and Histrionic subscale (eight items, e.g., “I am sexier than most”) were obtained by summing “false” (scored 0) or “true” (scored 1) responses, with a higher score indicating a greater level of traits associated with narcissistic and histrionic personality. Reliabilities were: narcissistic *α* = .63 and histrionic *α* = .47.

## Results

Nonverbal sexual coercion (35.2%) was the most commonly reported form of sexual coercion, followed by the use of emotional manipulation and deception (15.5%), and exploitation of the intoxicated (4.9%). As only one woman reported using physical force or threats, this subscale was not included in subsequent analyses. Correlation analyses (Table 1) demonstrated positive associations between the nonverbal sexual arousal form of sexual coercion, both pornography interest and effort, and HPD traits. Both the use of emotional manipulation and deception to coerce a partner and exploitation of the intoxicated were positively correlated with both pornography effort and compulsivity, and HPD traits. Additional correlations were identified between variables and between forms of sexual coercive behavior.

**Table 1:** Correlations between pornography interest, effort, and compulsivity, narcissistic and histrionic personality disorder traits, and sexual coercion

	POI	POE	POC	NPD	HPD	NVA	EMD	EXI
POI								
POE	.36**							
POC	.13	.38**						
NPD	.01	.15	−.05					
HPD	.04	.28**	.18*	.45**				
NVA	.17*	.27**	.06	.09	.22**			
EMD	.14	.38**	.24**	.12	.25**	.34**		
EXI	.11	.22**	.20*	−.02	.29**	.33**	.27**	
M	2.04	5.29	17.01	1.75	2.49	.58	.21	.06
SD	.18	.70	5.39	1.72	1.61	.93	.54	.26
Range	2–4	5–10	11–77	0–9	0–8	0–3	0–8	0–2

*POI* pornography interest, *POE* pornography effort, *POC* pornography compulsivity, *NPD* narcissistic personality disorder traits, *HPD* histrionic personality disorder traits, *NVA* nonverbal sexual arousal, *EMD* emotional manipulation and deception, *EXI* exploitation of the intoxicated

*p < .05, **p < .01

A series of multiple linear regressions were conducted to determine whether pornography interest, efforts, and compulsivity as well as NPD and HPD traits were predictors of sexual coercion (nonverbal sexual arousal, emotional manipulation and deception, and exploitation of the intoxicated) (see Table 2). The regression model was a significant predictor of nonverbal sexual arousal, *F*(5, 136) = 3.28, *p* = .008, explaining 10.8% of the sexual coercion variance (*R*^2^ = .11, Adj *R*^2^ = .08). Pornography effort was the only individual predictor significantly associated with this form of sexual coercion (*Β *=* .22, t *= 2.29, *p *= .024). A second regression revealed that the model was a significant predictor of emotional manipulation and deception, *F*(5, 136) = 5.83, *p* < .001, explaining 17.6%  % of the sexual coercion variance (*R*^2^ = .18, Adj *R*^2^ = .15). Pornography effort is the only significant individual predictor of emotional manipulation and deception (*Β *=* .29, t *= 3.14, *p *= .002). Finally, a third regression indicated that the model was a significant predictor of exploitation of the intoxicated, *F*(5,136) = 4.47, *p* = .001, explaining 14.1% of the sexual coercion variance (*R*^2^ = .14, Adj *R*^2^ = .11). HPD traits were the only significant individual predictor (*Β *=* .32, t *= 3.45, *p *= .001).

**Table 2:** Multiple linear regression results for pornography interest, effort, and compulsivity, narcissistic and histrionic disorder personality traits, and sexual coercion

Coercive behavior	ANOVA	*R*^2^	Adj *R*^2^	Individual predictor	*Β*	*t*	*p*
Nonverbal sexual arousal	*F*(5, 136) = 3.28, *p* = .008	.11	.08	Interest	.09	1.05	.295
				Effort	.22	2.29	.024
				Compulsivity	− .07	− .81	.421
				Narcissistic	− .03	− .29	.776
				Histrionic	.18	1.87	.063
Emotional manipulation and deception	*F*(5, 136) = 5.83, *p* < .001	.18	.15	Interest	.01	.17	.869
				Effort	.29	3.14	.002
				Compulsivity	.11	1.24	.217
				Narcissistic	.01	.14	.888
				Histrionic	.15	1.61	.111
Exploitation of the intoxicated	*F*(5, 136) = 4.47, *p* = .001	.14	.11	Interest	.05	.53	.596
				Effort	.11	1.15	.253
				Compulsivity	.08	.96	.337
				Narcissistic	− .17	− 1.93	.056
				Histrionic	.32	3.45	.001

## Discussion

Confirming expectations, pornography effort was associated with women’s use of the nonverbal sexual arousal and emotional manipulation and deception forms of sexual coercion. This finding is broadly consistent with the previous research that links women’s pornography use with a range of sexual coercive behaviors, such as harassment, verbal coercion, emotional manipulation, and deceit (Kernsmith & Kernsmith, 2009a; Wright et al., 2016), though additional research is required to consider why pornography interest and compulsivity were not associated with sexually coercive behavior. As there is little in terms of comparable research, explanations for these findings are proposed with caution. For example, as the previous research with male participants found compulsive pornography use to be related to the use of sexual coercion (e.g., Gonsalves et al., 2015), this disparity may reflect a sex difference. However, the alpha coefficients for the sexual compulsivity measures used in their study were low, confounding efforts to compare findings. As this area merits further exploration, it would be prudent for future studies to explore different elements of pornography use and sex differences further.

Our study also found that HPD traits were significantly associated with exploitation of the intoxicated, which the literature indicates may reflect excessive emotionality, demands for attention, and use of provocative behavior to manipulate others (e.g., AlaviHejazi et al., 2016; Bornstein & Malka, 2009; Dorfman, 2010; Stone, 2005). Indeed, women may be more likely to coerce a partner when feeling rejected (Wright, Norton, & Matusek, 2010). Unlike men (who are reportedly more likely than women to be motivated by power), sexually coercive women are reported to be motivated by affiliation–intimacy (Zurbriggen, 2000), which may be exaggerated in women with HPD traits who display increased sexual preoccupation (Apt & Hurlbert, 1994). The use of coercive behavior to sexually exploit the intoxicated could reflect the low levels of sexual assertiveness reported in women with HPD (see Apt & Hurlbert, 1994), thereby inhibiting the use of other forms of sexual coercion that require some degree of force. We did not observe the expected influence of NPD traits on sexual coercion. This was predicted due to previously reported associations between narcissism, sexual harassment (Zeigler-Hill et al., 2016), and coercion (Blinkhorn et al., 2015). This finding could also be indicative of similarities between NPD and HPD traits (as noted by Apt & Hurlbert, 1994; Widiger & Trull, 2007); thus, it would be advantageous for future investigations to explore this more explicitly.

As extant research is sparse and findings mixed, we did not make predictions on the use of physical force or threats to coerce a partner, and ultimately, as only one participant reported this, this subscale was excluded from analysis. Studies that do not include pornography use as a potential factor for sexual coercion report that women are less likely to use physical force or threats than they are to use other sexually coercive behavior, such as verbal pressure (Krahé et al., 2015), possibly indicative of greater caution or fear of retaliation. Indeed, female perpetrators of sexual coercion experience more negative reactions and resistance by victims than male perpetrators (O’Sullivan, Byers, & Finkelman, 1998). Yet, to complicate this further, studies that do examine the influence of pornography use on sexual coercion report contrary findings. For example, a meta-analysis of 22 studies found that women’s pornography use predicted all forms of sexual coercion, including physical force and threats (e.g., Wright et al., 2016), while another study found, to the contrary, that women’s pornography use was not associated with physical intimidation and force (e.g., Kernsmith & Kernsmith, 2009a). Future research could investigate these elements collectively to consider whether use of pornography influences women to employ physical force or threats only when other forms of sexual coercion fail, or if there are specific factors that explain the use of physical force and threatening behavior.

### Limitations and Further Research Directions

Despite efforts to recruit more participants, this study was limited by its use of a small, non-probabilistic sample; thus, generalizability is limited. As noted in other studies, the use of self-report questionnaire measures to investigate the sensitive topic of sexual coercion perpetration (e.g., Gonsalves et al., 2015) and personality disorder traits (Hoffmann & Verona, 2018; Khan et al., 2017; Muñoz et al., 2011) may have resulted in social desirability or bias recall. Further, the Cronbach’s alphas for some subscales were low. In part, this reflects the nature of the measure. (The exploitation of the intoxicated and pornography interest subscales contained two items each.) More extensive, detailed measures are recommended for future research. In particular, it was an oversight to overlook the potential influence of different types of pornographic materials, as women are exposed to a range of sexually explicit materials, including violent versus nonviolent pornography (Mattebo, Tyden, Haggstrom-Nordin, Nilsson, & Larsson, 2016). Pornography may contain violent or degrading scenes (Romito & Beltramini, 2015) or stereotyped depictions of females (Zhou & Bryant, 2016), which women are reportedly less aroused by than are men (Glascock, 2005). Important differences may also occur between amateur and professional pornography, with regard to the level of gender inequality featured (Klaassen & Peter, 2015). As important sex differences may occur with regard to the frequency and form of pornography use (Bohm, Franz, Dekker, & Matthiesen, 2015; Hald & Stulhofer, 2016), it would be useful for future studies to directly examine the influence of different types of pornography used by women on their sexually coercive behavior, rather than extrapolate from existing male-oriented research.

Despite efforts to recruit a diverse range of participants, the number of demographic items presented in the questionnaire was restricted, partly due to stringent ethical guidelines; thus, we were unable to examine racial differences in relation to sexual coercion. This may have been interesting to explore as a previous study has found that Asian males report significantly lower rates of sexual coercion victimization in comparison with their Black, White, and Latino counterparts (see French, Tilghman, & Malebranche, 2015). Other factors that previous studies report as significant mediating factors for sexual coercion in women, and thus are likely to yield valuable results in future research, include the influence of alcohol (Ménard et al., 2003) and sexual abuse history (Anderson, 1996; Russell & Oswald, 2001; 2002). Alcohol use may be of particular importance given that this study found HPD traits to be significantly associated with sexual exploitation of the intoxicated. To align with other general population research, this study aimed to examine sexually coercive behavior in women with no sex offense charges; despite recruiting participants from community and student populations, this caveat could only be inferred as questions to explicitly measure sex offending history were not included. Thus, future studies with females could directly measure participants’ engagement in criminality or could recruit participants with known sexual offending histories from clinical or forensic populations.

Females’ sexual coercion of males is often considered as less harmful by the general population than the same victimization of women by men (French et al., 2015; Huitema & Vanwesenbeeck, 2016; Struckman-Johnson et al., 2003; Studzinska & Hilton, 2017). Although male victims of female sexual coercion may also report positive responses to sexual coercion, some studies have reported that 90% of men also report at least one negative response to coercion (Kernsmith & Kernsmith, 2009b) and display significant psychological distress and risk behaviors (French et al., 2015; Turchik, 2012; Walker, Archer, & Davis, 2005). Relatively little research is available, however, to identify factors that influence attribution of blame to female perpetrators. Initial findings suggest that while male perpetrators are perceived to be aggressive, female perpetrators are regarded as promiscuous (Oswald & Russell, 2006). Additional research would be useful to determine factors that influence perceptions of victimization, victim-reporting or self-identification as a perpetrator or victim. An exploration of sexual coercion experienced by females who identify as LGBTQ is also a worthy avenue of further investigation, as previous studies note this may be prevalent but underreported (e.g., Turell, 2000; Waterman, Dawson, & Bologna, 1989). Finally, it is important to emphasize that the current study investigated women’s perpetration of sexually coercive behavior rather than men’s behavior after initial refusal. A range of individual and situational factors may predict responses to sexually coercive behavior such as persuasion that sexual activity is desirable, compliance with unwanted sex, or termination of a relationship (e.g., Nurius & Norris, 1996). The extent to which women’s sexually coercive behavior results in intercourse remains unclear, however, and future research could consider, for example, whether men experiencing sexual coercion subsequently engage in sex and the extent to which this is unwanted. Similarly, the present study did not assess women’s responses to their partner’s refusal. While it has been reported that women experience more negative reactions to sexual rejection than men (de Graaf & Sandfort, 2004), those factors impacting on responses to rejection remain unclear.

To conclude, we investigated factors associated with women’s use of sexual coercion. Findings indicate that women’s effort to use pornography was significantly associated with two subtypes of sexual coercion: nonverbal sexual arousal and emotional manipulative and deception to sexually coerce, while HPD traits were associated with exploitation of the intoxicated. Future research should further investigate the influence of pornography effort and HPD traits on aversive sexual behavior and the extent to which these may inform future intervention.

## Footnotes

1 “Trending” refers to a topic that experiences a surge in popularity for a limited duration of time, from which e-commerce businesses can extrapolate what is holding consumer interest.
